# Notes and Comments

**Published:** 1900-06

**Authors:** 


### Notes and Comments.[1]

Death under Nitrous Oxide Gas.—The following paragraph from a recent issue of the New York *Herald* tells the story of a death supposed to be due to the effects of nitrous oxide gas:

"George F. Terrell, master mechanic for the American Pin Company, of Waterbury, Conn., took his twelve-year-old son, Andrew, to the office of Dr. Virgil Munson, a dentist, this afternoon. The boy was suffering severely from an aching tooth—one of the big molars. He refused to submit to the operation without an anæsthetic, and was given nitrous oxide, known as laughing gas. The tooth came easily, but the boy died within a few minutes. The

[1] The assistant editor solicits contributions for this department,—new methods, new remedies and formulas, or any short practical note which may prove of value to the practitioner or student. Address 1338 Walnut Street, Philadelphia.

doctor says there was apparently no physical reason why the youth could not stand the anæsthetic, but friends of the boy are dissatisfied."

---

On the Advance in Dental Educational Requirements.—In the *Items of Interest,* Dr. L. Ashley Faught very well says that to the close student of the history of dental instruction in the United States it is apparent that we are entering to-day upon a new era,—that in which a sharp distinction will be drawn between the general development of the understanding and the special training necessary for the following of a special pursuit. A third stage in our career has been reached. It is not a new creation,—not even a new beginning,—but an evolution from what has preceded it.

Briefly stated, the stages are these: A chaotic period reaching from the inception of the profession up to about 1890 of heterogeneous and indefinite educational entrance requirements. Then for nearly a decade, up to 1899, a second period, marked by the struggle to establish a homogeneous and, as progress was made, a high and definite qualitative educational entrance requirement. The third stage,—that of to-day,—the working out of the qualitative factor.

---

Perfection in Dental Operations.—Dr. Grant Mitchell, in the *Dental Brief,* says,—

"Even among the students who imagine that gold fillings constitute the dizziest heights of dental possibilities, the majority succeed in mastering only the meagre rudiments of their proper introduction, and fail to grasp, in their fulness, as in their refinements, the underlying principles of that department of practice; they do things thus and so because the demonstrator told them to! Failing to get at the 'reason why,' they never come to an understanding of the all too evident fact that dentistry offers no middle ground upon which to stand; that nothing may be effected which will merely 'answer the purpose;' that operations are accomplished perfectly, or they are simply imperfect; and that there cannot exist a 'slight' imperfection in any dental operation."

Remedies for the Office.—In reply to an inquiry from a "recent graduate" as to the best stock of medicines for one in fitting up an office, we cannot do better than give the advice offered by Dr. Welch in writing upon the subject. He said it was much better to have a few good familiar remedies on hand than many that you are in doubt about, or that you are experimenting with. So with your treatment of a given class. Make a study of them till you are clearly satisfied with what you should do, and then do it. Continually varying your remedies and your practice is confusing, and tends to indecision and unreliable practice. Not that we should never take on anything new, or never discard anything old, or never vary our practice, but have some things settled, and as many things settled as possible, and then in investigating new things or processes go at it deliberately, systematically, and intelligently, and settle that. This never knowing and being forever coming to a knowledge of the truth is vexatious, bewildering, and childish.

